# Early screening for *Chlamydia trachomatis* in young women for primary prevention of pelvic inflammatory disease (i-Predict): study protocol for a randomised controlled trial

**DOI:** 10.1186/s13063-017-2211-1

**Published:** 2017-11-13

**Authors:** Jeanne Tamarelle, Anne C. M. Thiébaut, Bénédicte Sabin, Cécile Bébéar, Philippe Judlin, Arnaud Fauconnier, Delphine Rahib, Layidé Méaude-Roufai, Jacques Ravel, Servaas A. Morré, Bertille de Barbeyrac, Elisabeth Delarocque-Astagneau, i-Predict study group, Elisabeth Delarocque-Astagneau, Anne Thiébaut, Jeanne Tamarelle, Bertille de Barbeyrac, Cécile Bébéar, Servaas Morré, Jacques Ravel, Emmanuelle Mathiot-Vicaigne, Christian Régnier, Philippe Aoussou, Raphaelle Badie-Perez, Karine Rebouillat, Christophe Tzourio, Anne-Cécile Rahis, Arnaud Fauconnier, Antoine Bourret, Jean-Luc Brun, André Bongain, Philippe Aegerter, Layidé Méaude-Roufai, Delphine Rahib, Nathalie Lydié

**Affiliations:** 1 Biostatistics, Biomathematics, Pharmacoepidemiology and Infectious Diseases (B2PhI), Inserm, UVSQ, Institut Pasteur, Université Paris-Saclay, 2 avenue de la source de la Bièvre, 78180 Montigny-le-Bretonneux, France; 2 French National Reference Centre for Chlamydia, USC EA 3671, Mycoplasmal and Chlamydial Infections in Humans, University of Bordeaux, Campus Bordeaux Carreire, 146 rue Léo Saignat, 33076 Bordeaux cedex, France; 3 Service de Gynécologie Obstétrique, Centre Hospitalier Régional Universitaire de Nancy, 10 rue du Dr Heydenreich, 54000 Nancy, France; 4 Research Unit EA 7285, Risk and safety in clinical medicine for women and perinatal health, Versailles-Saint-Quentin University (UVSQ), Montigny-le-Bretonneux, France; 5 Department of Gynaecology and Obstetrics, Intercommunal Hospital Centre of Poissy-Saint-Germain-en-Laye, Poissy, France; 6 Santé Publique France, 12 Rue du Val d’Osne, 94410 Saint-Maurice, France; 7 Department of Clinical Research, URC HUPIFO, Hôpital Ambroise Paré, Assistance Publique – Hôpitaux de Paris (AP-HP), 9 avenue Charles de Gaulle, 92100 Boulogne-Billancourt, France; 8 Institute for Genome Sciences, University of Maryland School of Medicine, 801 West Baltimore Street, Baltimore, MD 21201 USA; 9 Department of Microbiology and Immunology, University of Maryland School of Medicine, 801 West Baltimore Street, Baltimore, MD 21201 USA; 10 Laboratory of Immunogenetics, Department of Medical Microbiology and Infection Control, Research School V-ICI, VU University Medical Centre, De Boelelaan 1118, 1081HV Amsterdam, The Netherlands; 11 Institute for Public Health Genomics, Department of Genetics and Cell Biology, Research Institute GROW (School for Oncology and Developmental Biology), Faculty of Health, Medicine and Life Sciences, University of Maastricht (UM), Universiteitssingel 50, 6229 ER Maastricht, The Netherlands

**Keywords:** *Chlamydia trachomatis*, Infection, Clearance, Reinfection, Screening, Pelvic inflammatory disease, Prevention, Students, Immunogenetics, Natural course of infection

## Abstract

**Background:**

Genital infection with *Chlamydia trachomatis* (Ct) is the most common bacterial sexually transmitted infection, especially among young women. Mostly asymptomatic, it can lead, if untreated, to pelvic inflammatory disease (PID), tubal factor infertility and ectopic pregnancy. Recent data suggest that Ct infections are not controlled in France and in Europe. The effectiveness of a systematic strategy for Ct screening in under-25 women remains controversial. The main objective of the i-Predict trial (Prevention of Diseases Induced by *Chlamydia trachomatis*) is to determine whether early screening and treatment of 18- to-24-year-old women for genital Ct infection reduces the incidence of PID over 24 months.

**Methods/design:**

This is a randomised prevention trial including 4000 eighteen- to twenty-four-year-old sexually active female students enrolled at five universities. The participants will provide a self-collected vaginal swab sample and fill in an electronic questionnaire at baseline and at 6, 12 and 18 months after recruitment. Vaginal swabs in the intervention arm will be analysed immediately for Ct positivity, and participants will be referred for treatment if they have a positive test result. Vaginal swabs from the control arm will be analysed at the end of the study. All visits to general practitioners, gynaecologists or gynaecology emergency departments for pelvic pain or other gynaecological symptoms will be recorded to evaluate the incidence of PID, and all participants will attend a final visit in a hospital gynaecology department. The primary endpoint measure will be the incidence of PID over 24 months. The outcome status (confirmed, probable or no PID) will be assessed by two independent experts blinded to group assignment and Ct status.

**Discussion:**

This trial is expected to largely contribute to the development of recommendations for Ct screening in young women in France to prevent PID and related complications. It is part of a comprehensive approach to gathering data to facilitate decision-making regarding optimal strategies for Ct infection control. The control group of this randomised trial, following current recommendations, will allow better documentation of the natural history of Ct infection, a prerequisite to evaluating the impact of Ct screening. Characterisation of host immunogenetics will also allow identification of women at risk for complications.

**Trial registration:**

ClinicalTrials.gov, NCT02904811. Registered on September 14, 2016.

World Health Organisation International Clinical Trials Registry, NCT02904811.

AOM, 15-0063 and P150950. Registered on September 26, 2016. A completed Standard Protocol Items : Recommendations for International Trials (SPIRIT) Checklist is available in additional file 1.

**Electronic supplementary material:**

The online version of this article (doi:10.1186/s13063-017-2211-1) contains supplementary material, which is available to authorized users.

## Background

### Epidemiology of genital infections with *Chlamydia trachomatis* and further complications

Genital infection with *Chlamydia trachomatis* (Ct) is the most common bacterial sexually transmitted infection (STI) [1]. Ct affects predominantly young women (18–24 year old), with an estimated prevalence of 3.6% in 2005 in France [2]. The number of Ct diagnoses has increased over the past 10 years in France as well as in other European countries. Interestingly, in 2012, the proportion of positive tests reached 8.3% of 18- to 24-year-old women participating in a web-based study promoting home screening in France [3]. Although these trends may be partly explained by an increase in screening activities, particularly targeting at-risk populations, they may reflect a true increase in incidence.

Because the majority (~70–80%) of lower genital tract Ct infections in women remains asymptomatic [4], patients may not seek testing for care. If untreated, genital chlamydial infection can ascend to the upper genital tract, leading to serious complications [5] such as pelvic inflammatory disease (PID), tubal factor infertility [6] and ectopic pregnancy [7, 8].

### Prevention strategies in France and in other European countries

Early diagnosis and antibiotic treatments have been considered a major strategy for prevention of complications and further transmission of Ct infection. In France, the national chlamydia control strategy includes sexual health education, awareness campaigns, promotion of condoms and at-risk population screening. Systematic (routine) screening in young sexually active women has not been implemented. Instead, opportunistic screening for Ct infection is offered to women younger than 25 years old if they visit STI clinics, reproductive health services or abortion centres [9]. A systematic screening programme was introduced throughout England in 2007 for sexually active men and women younger than 25 years old attending various clinical and non-clinical settings (e.g., universities and sporting events), but the effectiveness of systematic screening to prevent PID remains controversial.

### Gaps in knowledge

To date, there have been three published randomised trials in which researchers investigated whether early screening and treatment of young females would reduce the incidence of PID over a 12-month period [10–12]. The most recent and methodologically sound study, the prevention of pelvic infection (POPI) trial conducted in England, yielded inconclusive results. Moreover, most PID cases occurred in women who had a negative test result for Ct at baseline, raising the question of the optimal frequency of testing [12].

Key characteristics of Ct infection natural history (i.e., rate and timing of progression of lower genital tract infection to PID) are not sufficiently documented. Yet, accurate estimates of these characteristics are needed to better anticipate the benefit of early and systematic screening for infection. Further, the average duration of Ct infection in the absence of treatment is not well established and could possibly be 1 year or longer [13]. Finally, reinfection is common following therapy (10–20% patients within 12 months) [14, 15], thus raising questions about host immune response and the relevance of and time to retesting.

Inter-individual variations in host immune response may explain why some women develop complications such as PID after Ct infections and others do not. Among various genetic markers, single-nucleotide polymorphisms (SNPs) have been shown to be related to the development of late complications of Ct infections in women [16, 17]. This approach could be relevant to identifying women at risk for PID and tubal pathology and adapting treatment and follow-up for these women. In summary, there is a need to evaluate the efficacy of early screening for prevention of PID and to better understand the natural history of Ct infection and progression to PID in order to develop efficient screening programmes in France.

### Main objective

The main objective of this study is to determine whether early screening and treatment for genital Ct in young women (<25 years of age) in France reduces the cumulative incidence of PID over 24 months.

### Secondary objectives

The following are secondary objectives of this study:

- To determine the baseline prevalence and the incidence of Ct infection
- To improve knowledge of the natural history of Ct infection in young women, including rate and timing of progression to PID, as well as the incidence of Ct reinfection
- To investigate the relationship between host immunogenetics (SNPs), Ct clearance, persistence, and development of late complications (PID) as an explanatory factor for inter-individual heterogeneity in Ct susceptibility and infection progression.

To reach these objectives, the i-Predict trial (Prevention of Diseases Induced by *Chlamydia trachomatis*) leverages the existing i-Share cohort (Internet-based Students HeAlth Research Enterprise), the largest French prospective cohort, aiming at recruiting 30,000 students followed for at least 10 years with yearly assessments of their health. Participants in i-Share are asked to complete baseline and follow-up web-based questionnaires through a dedicated study website (www.i-share.fr). The objective of i-Share is to evaluate the frequency of diseases affecting students and related risk factors. It is also meant to serve as a platform to nest interventional studies on specific topics related to students’ health.

## Methods/design

### Design/setting

**Fig. 1:**
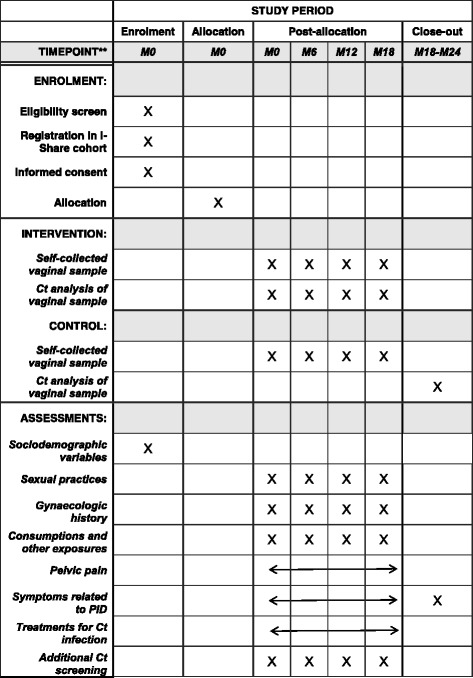
Design of the i-Predict trial according to the SPIRIT checklist. *Ct Chlamydia trachomatis*

This is a randomised controlled trial spanning 24 months. It is a single-blinded study nested within the i-Share cohort study (Fig. 1).

### Participants

- Inclusion criteria
  - Female student registered at a French university participating in the i-Share cohort
  - Aged 18 to 24 years
  - Sexually active
  - Signed written informed consent
  - Covered by the French National Health Insurance programme
- Non-inclusion criteria
  - Reported pregnancy

### Intervention

#### Experimental group

Vaginal samples from the intervention group (collected at months 0, 6, 12 and 18) will be tested immediately for Ct infection. Participants with positive results for Ct will be asked to attend a visit with their general practitioner or gynaecologist or at the nearest STI clinic to be examined and treated, and they will be informed of the need for their partner to be treated (‘patient referral’ is acceptable in this French setting).

#### Control group

The control group will perform self-taken vaginal samples at the same frequency as the intervention group, but testing will be deferred until the end of the 18-month study period to allow study of the natural course of Ct infection in the absence of testing and treatment. Results for Ct will be made available to the gynaecologist at the final visit for adequate care. During the whole study, participants will follow current screening guidelines (i.e., screening in case of symptoms or if they attend STI clinics).

### Blinding

Participants will be blinded to their group assignment until the end of the trial except for those who will be informed of a positive result in the intervention group. Two independent experts will review symptoms data from medical records and symptoms collected during the final visit to classify outcome status (confirmed, probable or no PID). These medical experts will be blinded to group assignments and Ct status.

### Recruitment, randomisation, follow-up and data collection

**Fig. 2:**
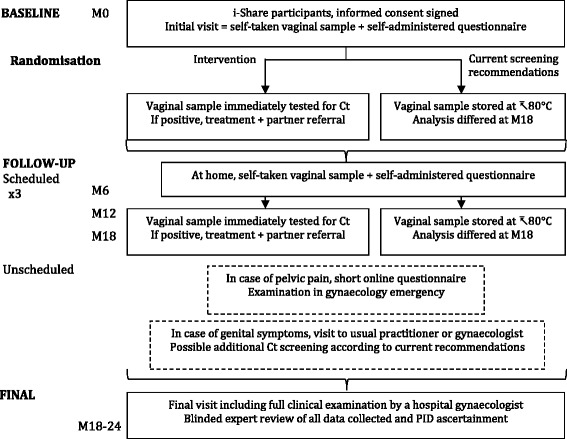
Schedule of enrolment, interventions and assessments of the i-Predict trial. *Ct Chlamydia trachomatis*, *PID* Pelvic inflammatory disease

i-Predict is nested within the i-Share cohort; therefore, information provided to the students in the i-Predict trial will be available on the i-Share website, redirecting volunteers to the university health service (UHS) to be included. Participants in i-Share who fit the inclusion criteria for i-Predict (age and sex) will be solicited via emails to participate. Participants can also be informed about i-Predict and asked to participate at each UHS of the five participating universities without knowledge of the existence of the i-Share cohort or solicitation through the i-Share cohort. In this case, they will also be enrolled in the i-Share cohort just before the i-Predict initial visit. A schedule of enrolment, interventions and assessments of the i-Predict trial is shown in Fig. 2.

#### Initial visit

Female students will be enrolled at each UHS for a period of 2 years. They may already be enrolled, or they may be new participants in the i-Share cohort (i.e., enrolled during the visit at which they will be offered to participate in both the i-Share cohort and the i-Predict trial).

Eligible participants will be approached by the physician investigator, provided information on the study and asked to sign an informed consent form if they agree to participate. They will be randomly allocated to the intervention or control group.

Participants will complete a baseline electronic self-administered questionnaire on their sexual and gynaecological history, use of hormonal contraception and condoms, and possible symptoms of infection. They will also provide a self-collected vaginal sample (month 0) on site.

Additionally, the following sociodemographic and medical data from the i-Share baseline questionnaire will be retrieved at baseline: type of training; year of registration; dwelling place; medical history; medical appointments; medication consumption; eating habits; well-being; and tobacco, alcohol and drug use.

#### Randomisation

Block randomisation, stratified by participating university (Versailles Saint-Quentin-en-Yvelines, Bordeaux, Nice Sofia-Antipolis, Paris Sorbonne Universités, and Sorbonne Paris Cité), will be used to assign eligible participants who have provided informed consent to each group (1:1 ratio), resulting in one intervention group and one control group. Site-specific randomisation will be performed in the UHS of each of the five participating universities by the principal investigator at each site on the Cleanweb® electronic platform as soon as student recruitment is registered during the initial visit. Group assignment is generated at the same time as the participant’s study identification number; only principal investigators at each site and dedicated staff from the Centre National de Référence (French National Reference Centre for Chlamydial Infections) (CNR) have access to group assignment.

#### Follow-up

All participants will self-collect vaginal samples in the privacy of their home at months 6, 12 and 18. A kit including detailed self-sampling instructions will be mailed to them a few days prior to each due date. Participants will mail the sample back to the CNR and complete an electronic self-administered questionnaire about their number of sexual partners, condom use, symptoms such as abnormal vaginal discharge or pelvic pain, additional testing since their previous sample, medical visits and antibiotic use (for Ct or other infections).

Participants with a positive test result for Ct at baseline or months 6, 12 or 18 in the intervention group will be referred to their physician or the nearest STI clinic for treatment. Of note, current recommendations for uncomplicated infections include a single-dose therapy of azithromycin (1 g) or a 7-day course of doxycycline [18].

The physician collecting clinical data upon examination will fill out a specific form, handed out by the participant together with a prepaid envelope, on clinical findings, as well as treatment initiated, results of additional tests or ultrasound (if any), and information regarding whether a partner was treated (if available). These data will be received at the UHS and entered by a trained clinical research assistant on the Cleanweb® platform.

At any moment during follow-up, all participants will be asked to complete a short, self-administered questionnaire online each time they feel unusual lower abdominal pain. A contact will be made with the participant 24–48 h after completion of this short pelvic pain questionnaire to encourage the participant to visit the closest participating gynaecology emergency department (GED), where they will be evaluated for PID according to current national recommendations by the Collège National des Gynécologues et Obstétriciens Français [19]. Data from these visits to the GED will be made available to the trained clinical research assistant, who will collect them using a standardised clinical research form and enter the clinical data on the Cleanweb® platform.

Unscheduled visits to general practitioners and gynaecologists for gynaecologic symptoms are expected to occur, during which participants may receive a diagnosis of PID. Clinical data from these consultations will be collected using a specific form filled out by the physician and handed out by the participant together with a prepaid envelope (clinical examination, treatments, results of additional tests or ultrasound if any). This form will be received at the UHS, and data will be entered on the Cleanweb® platform by a trained clinical research assistant. During the follow-up phase, participants will be reminded by email or by phone to collect the swab and fill out their questionnaires.

#### Final visit

The final visit will take place between months 18 and 24 (depending on enrolment date to avoid the possibility that the final visit takes place during university summer break) and will include an extensive clinical examination by a gynaecologist for signs suggestive of PID and, if indicated, pelvic ultrasound. The gynaecologist will have access to chlamydia test results for both groups. For participants in the control group, the gynaecologist will review Ct results throughout the course of the study and decide whether the participant should get treatment based on these results. Final visits will take place at the hospital gynaecology department in geographical proximity to the recruiting universities: Poissy/Saint-Germain-en-Laye Hospital, Nice L’Archet Hospital, Paris Cochin Hospital and Bordeaux Pellegrin Hospital.

In the case where participants do not complete follow-up, attempts will be made to obtain information on the reasons for dropout. The last contact date will be recorded. To compensate participants for the time dedicated to the study, financial compensation will be given at the initial visit and at the final visit.

### Outcomes

#### Primary endpoint

The primary endpoint measure is the cumulative incidence of first PID over 24 months in the intervention group (immediate testing and treatment) and in the control group following current screening recommendations (deferred testing). PID comprises a spectrum of inflammatory disorders of the female upper genital tract, including endometritis (inflammation of the uterine lining), salpingitis (inflammation of one or both fallopian tubes), tubo-ovarian abscess and pelvic peritonitis.

For the purpose of this study, the definition of PID is as follows [20]:

- *Probable PID:* Clinical diagnosis of PID (pelvic pain, cervical motion tenderness, uterine or adnexal tenderness)
- *Confirmed PID:* Clinical diagnosis of PID and one of the following criteria: laparoscopic abnormalities consistent with PID, endometrial biopsy with histopathologic evidence of endometritis, pelvic ultrasound showing thickened tubal wall (>8 mm), tubal fringes or free pelvic fluid

Data on symptoms reported by the participants and collected through medical records, as well as data collected at the final visit, will be reviewed by two independent experts blinded to group allocation (intervention or control) and to Ct infection status to classify the outcome status (no PID, probable PID or confirmed PID).

#### Secondary endpoints

The following secondary endpoints will be measured:

- Prevalence of Ct infection in participants at baseline (intervention and control groups)
- Incidence of first Ct infection in participants with a negative Ct test result at baseline (intervention and control groups)
- Incidence of first PID (intervention and control groups)
- Duration of Ct infection (6 or 12 months or over 18 months because the time window between each assessment is 6 months); proportion of infections associated with a diagnosis of PID (whether at a previous visit or concurrently) and time to development of PID since Ct infection; proportion of spontaneous Ct infection resolution (control group), defined as a Ct-negative sample after one or more Ct-positive samples
- Incidence of reinfections (intervention and control groups); an algorithm to differentiate between Ct reinfection, treatment failure and persistent infection [21] will be developed on the basis of typing (as described below in laboratory analyses), treatment, partner notification and change of partner

### Materials/laboratory analyses

**Fig. 3:**
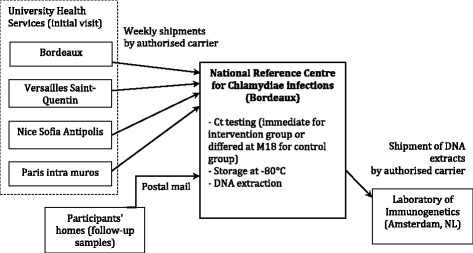
Sample flow of the i-Predict trial

Non-invasive screening options (i.e., self-collected vaginal swabs, avoiding speculum examination) have been developed and validated in the last decade [22] and have high acceptability [23]. Participants will use commercially available Conforme aux Exigences (CE)-marketed Aptima Multitest swab specimen collection kits (Hologic, San Diego, CA, USA) to provide their vaginal samples at months 0, 6, 12 and 18. The flow of the trial is depicted in Fig. 3.

After sampling, the kit will be returned to the CNR (Bordeaux, France) in a pre-addressed, prepaid envelope. At the CNR, samples will be recorded. All samples will be analysed using a commercially available, CE-marketed, transcription-mediated amplification assay (Aptima Combo 2 CT/NG; Hologic) on the automated Panther instrument (Hologic), which performs well on self-collected vaginal swabs [24]. In the intervention group, samples will be analysed upon arrival. Analysis will be deferred for the control group to after the 18-month study period, and the samples will be stored at − 80 °C immediately after being received.

To determine whether a Ct infection is persistent or new, typing methods developed in the CNR will be applied, enabling high-resolution molecular epidemiological characterisation of Ct genovars D to K: *omp1* gene PCR-restriction fragment length polymorphism [25] or *omp1* gene sequencing, followed by multiple loci variable-number tandem repeat analysis [26] in case the same genovar is identified in two samples from the same subject. The CNR will perform DNA extraction from vaginal swabs and transfer the prepared samples to the immunogenetics laboratory for SNP biomarker analyses. These will be performed using a set of over 50 SNPs already identified and validated to be predictive of tubal factor infertility susceptibility [16].

### Sample size

The cumulative number of PID cases (from baseline to up to 18 months) was estimated in each group, assuming no natural clearance of Ct infection in the absence of treatment, because Ct infection is mostly asymptomatic and the follow-up relatively short in comparison to infection duration, which is most likely > 1 year [13]; 100% treatment efficacy to clear Ct and PID; and an average 3-month lag between Ct acquisition and PID onset [27, 28]. The same number of Ct-infected and PID cases is expected in the two groups at baseline, assuming that observed Ct infections at baseline have been prevalent for an average of 9 months (hence a PID cumulative incidence over 6 months).

Assuming a 5% yearly incidence of Ct infection [14] and a 1% yearly incidence of PID among Ct-negative young women, we calculated that a sample size of 2000 participants in each group would allow us to detect a 0.54 relative risk (RR) between the intervention and control groups with a 5% one-sided type I error rate and 80% power, assuming either 5% Ct prevalence at baseline [12] and 12% PID incidence among Ct-positive participants or 7% Ct prevalence at baseline [29] and 10% PID incidence among Ct-positive participants [12], and accounting for a 10% attrition rate. Therefore, 4000 sexually experienced female students aged 18 to 24 years participating in the i-Share French student cohort will be enrolled.

### Statistical analyses

For the primary analysis, we will calculate the cumulative incidences of developing PID over 24 months in both groups and estimate the RR of developing PID as the ratio of these incidences (intention-to-treat analysis). We will consider probable and confirmed PID together first and conduct confirmatory analysis restricted to confirmed PID only.

For comparison with previous trials, we will further stratify our RR analysis on baseline status of Ct infection. We will also conduct an exploratory Cox regression analysis to adjust the RR of developing PID for other baseline characteristics of the participants. Missing data imputation will be considered for participants’ characteristics (excluding Ct and PID status) when missing values exceed 10%.

As secondary analyses, we will determine the following:

- Annual incidence of Ct infection based on the first recorded Ct infection among participants Ct-negative at baseline in both groups
- Among participants in the control group with incident Ct infection (participants Ct-negative at baseline), proportions (and 95% CIs) of Ct infections with spontaneous resolution and of Ct infections evolving towards PID; estimation of the duration of Ct infection (from Ct detection to clearance) and time to develop PID (time between incident Ct infection and incident PID, defined as from 3 months before Ct detection because time windows correspond to 6 months, to the onset of PID symptoms)
- Among participants in both groups who have cleared Ct infection at least once, incidence of Ct reinfection and comparison between the treated (intervention) and untreated (control) groups
- Among participants in the control group, exploratory logistic regression to assess whether 50 immunogenetic biomarkers can predict Ct persistence and occurrence of PID

### Trial governance

#### Sponsor

The i-Predict trial is sponsored by Assistance Publique – Hôpitaux de Paris (AP-HP, Département de la Recherche Clinique et du Développement). AP-HP is involved in the implementation of the trial, legal/ethical submissions and hosting the i-Predict database. It is not involved in the design of the study or in the analysis or interpretation of the data. AP-HP performs regular quality control assessments. A clinical research assistant will visit participating centres (UHS and GED) every 6 months to ensure that implementation is in accordance with the protocol. AP-HP has taken out insurance from HDI-Gerling through BiomedicInsure for the full research period, covering its own civil liability and that of any agent (doctor or research staff), in accordance with article L.1121-10 of the French Public Health Code.

#### Coordinating unit

AP-HP and the UMR 1181 are responsible for coordinating this trial. They will ensure that recruitment and follow-up are performed as planned. UMR 1181 is responsible for the statistical analysis of primary and secondary outcomes.

#### Investigating centres

UHS and GED are involved in the recruitment, inclusion and follow-up of participants. They collect informed consent from the participants and implement randomisation. They are responsible for notifying the sponsor’s vigilance division of any adverse event by filling in the adverse event section of the electronic case report form (Cleanweb® electronic platform).

#### Steering committee

The steering committee comprises coordinating investigators, scientific experts, a methodologist and biostatistician, and representatives of the sponsor, and it will meet twice yearly. The steering committee will be responsible for inquiring about research progress, monitoring compliance with good clinical practice and patient safety, deciding on any relevant modification of the protocol, and monitoring compliance with the rules of communication and publication of the results described in the protocol. Monthly conference calls will also be held to monitor recruitment completion and follow-up rates and to review specific issues raised by the participating centres.

#### Data monitoring committee

There will be no data monitoring committee, owing to low risks expected in this study.

## Discussion

### Expected results

The increasing prevalence of Ct in young women and recent improvements in the diagnosis of Ct infection (including availability of reliable self-taken devices) support reconsidering current screening recommendations for Ct infection. However, systematic screening as a strategy to lower the rate of PID needs to be evaluated. Further, better understanding of the natural history of Ct infection and progression to PID is required to develop a well-adapted screening programme. Systematic screening in young sexually active women has not been implemented in France, which provides an opportunity to conduct a community-based randomised trial to evaluate the efficacy of early Ct screening and treatment in preventing PID. The planned length of follow-up (24 months), the repeated Ct testing (every 6 months) and the control group will not only provide valuable insight into the clinical course of Ct infection but also create a unique opportunity to investigate host immunogenetic factors to explain inter-individual heterogeneity and ultimately to develop a tool to identify women at risk.

This trial will also serve as a platform for ancillary studies. An ancillary study on the role of the vaginal microbiota in facilitating or preventing Ct infection is planned. The vaginal microbiota of each participant throughout the study period will be characterised using culture-independent molecular methods relying on the sequencing of 16S ribosomal RNA gene amplicons as previously reported in other settings [30].

### Practical or operational issues

The target population is 18- to 24-year-old women because they have the highest incidence of Ct infection in the general population and because screening programmes implemented in other European countries (systematic or opportunistic, i.e., in STI clinics) target this age group. Our study population is, however, restricted to students. In France they represent a large part of this age group. This choice represents an issue in terms of the generalisability of our results. However, this choice was motivated by the fact that UHS have seen their missions reinforced in terms of prevention and care and are likely to play a role in offering Ct screening in the future, including for non-student females. In fact, four of five UHS have already or are in the process of becoming health centres (not limited to students, but localised in universities and with a special mission towards the student population). Another issue regarding the generalisability of our results is the enrolment of voluntary participants, which is inherent to a clinical trial. We limited the impact of this by creating a screening log of young women who declined to participate (age, university).

Because an aim of the i-Predict trial is testing a community screening strategy among a student population that is usually difficult to capture, the sample size necessary to reach our goal is substantial, and we anticipate difficulties in the recruitment and follow-up of our participants. To achieve our enrolment goal, we are leveraging the existing i-Share cohort and universities’ health care facilities. We therefore developed communication tools specific to our study population (website, social networks, flyers and specific events) to facilitate recruitment, which will also benefit from the existing i-Share cohort. We chose to use an electronic platform to automatically generate reminders before and after each follow-up due date. The participants will benefit from close follow-up by trained clinical research assistants. Participants will receive financial compensation for the time they dedicate to the study, in particular for the time-intensive initial visit at the UHS and for the final visit at the participating GED.

Ethically, the deferred Ct analysis of collected vaginal samples until month 18 in the control group represented a challenge. However, the aim of the i-Predict trial is testing a systematic screening strategy and comparing it with the current strategy in France, which is to propose Ct testing in women younger than 25 years old in STI clinics, family planning and on specific indications by the general practitioner or gynaecologist. Therefore, all participants are given information on current guidelines for Ct screening. They are also given detailed information on chlamydia and the potential long-term complications of the infection, and they are encouraged to follow current guidelines for Ct screening and get tested whenever they wish [9]. Finally, all participants will benefit from a full gynaecological examination at the final visit and from treatment if necessary. Because participants in the control group are given information, they are more aware of the issues related to Ct infection and screening than the general population. This additional information might result in less statistical power to demonstrate the efficacy of systematic screening, but it was ethically unquestionable. The choice of the comparator group was discussed and maintained because it allows answering key questions on the natural course of Ct infection in the absence of any treatment. This trial will be the first large-scale test in Europe allowing determination of whether screening and treating young women decreases the risk of PID as well as documentation of key characteristics of Ct natural history (Additional file 1).

## Trial status

The first enrolment date was January 10, 2017. The estimated last enrolment date will be in June 2019. The estimated follow-up completion date is January 2021. The estimated primary analysis completion date is October 2021.

## Additional file

Additional file 1: Standard Protocol Items: Recommendations for Interventional Trials (SPIRIT) 2013 checklist: recommended items to address in a clinical trial protocol and related documents. (DOC 53 kb)

## Abbreviations

AP-HP: Assistance Publique – Hôpitaux de Paris
CE: Conforme aux Exigences
Ct: *Chlamydia trachomatis*
CNR: Centre National de Référence (French National Reference Centre for Chlamydial Infections)
GED: Gynaecologic emergency department
i-Predict: Prevention of Diseases Induced by *Chlamydia trachomatis* trial
PID: Pelvic inflammatory disease
POPI: Prevention of pelvic infection trial
RFLP: Restriction fragment length polymorphism
RR: Relative risk
SNP: Single-nucleotide polymorphism
STI: Sexually transmitted infection
UHS: University health service
